# Single‐cell RNA sequencing of peripheral blood mononuclear cells from bronchopulmonary dysplasia

**DOI:** 10.1002/ctm2.70276

**Published:** 2025-03-17

**Authors:** Yufeng Liu, Chun Yan, Yushan Li, Ruoxing Zhou, Xiaoyu Lin, Qiong Meng, Sitao Li, Limei Zhong, Yanfang Tan, Wangkai Liu

**Affiliations:** ^1^ Center for Medical Research on Innovation and Translation Guangzhou First People's Hospital the Second Affiliated Hospital of South China University of Technology Guangzhou China; ^2^ Department of Pediatrics Guangdong Women and Children Hospital Guangzhou China; ^3^ Department of Pediatrics, the First Affiliated Hospital Sun Yat‐sen University Guangzhou China; ^4^ Department of Pediatrics Children's Hospital of Fudan University Shanghai China; ^5^ Center for Translational Medicine The First Affiliated Hospital Sun Yat‐sen University Guangzhou China; ^6^ Department of Pediatrics The Second People's Hospital of Guangdong Province Guangzhou China; ^7^ Department of Pediatrics The Sixth Affiliated Hospital Sun Yat‐sen University Guangzhou China; ^8^ Department of Laboratory Medicine Guangdong Second Provincial General Hospital Guangzhou China

## Abstract

**Background:**

Bronchopulmonary dysplasia (BPD) is a severe respiratory disease that primarily affects premature infants, characterized by persistent inflammation and abnormal immune activation. This study aimed to elucidate the immunological mechanisms underlying BPD by integrating single‐cell RNA sequencing with T/B cell receptor profiling of peripheral blood mononuclear cells (PBMCs) from preterm infants with BPD, complemented by validation in a murine BPD model.

**Methods:**

We profiled PBMCs from preterm infants diagnosed with BPD and healthy controls, identifying 22 distinct cell clusters corresponding to major immune cell types.

**Results:**

Significant alterations were observed in myeloid and lymphoid subsets, with neutrophils undergoing metabolic reprogramming toward oxidative phosphorylation. T and B cell subsets exhibited phenotypic and functional changes, with B cells serving as crucial interaction hubs in cell communication networks. Progenitor cell analysis in BPD mouse models revealed specific alterations in hematopoietic stem cells. Analysis of cell–cell communication networks highlighted intricate intercellular interactions in BPD, emphasizing a pivotal role for the BTLA‐TNFRSF14 signaling axis in disease pathogenesis. Additionally, pharmacological blockade of BTLA in mouse models alleviated disease severity, suggesting its potential therapeutic effects through modulation of the BTLA‐TNFRSF14 pathway.

**Conclusion:**

These findings enhance the understanding of the BPD immune microenvironment and lay the foundation for developing targeted immunomodulatory therapies.

**Highlights:**

- Single‐cell sequencing revealed immune cell profiles in bronchopulmonary dysplasia (BPD).
- Neutrophils underwent metabolic changes, and B cells were key in immune communication.
- Targeting B and T lymphocyte attenuator (BTLA)‐TNFRSF14 signalling reduced BPD severity in mouse models, suggesting a potential therapy.

1

Dear Editor,

Bronchopulmonary dysplasia (BPD) is a severe respiratory disease that primarily affects premature infants, characterized by persistent inflammation and abnormal immune activation.^1^ This study aimed to elucidate the immunological mechanisms underlying BPD by integrating single‐cell RNA sequencing (scRNA‐seq) with T/B cell receptor (TCR/BCR) profiles of peripheral blood mononuclear cells (PBMCs) from preterm infants with BPD, further validated in a murine BPD model.

We used scRNA‐seq to characterize PBMCs from four BPD patients and four healthy controls (Table S1), performed flow cytometry (FCM) analysis and established BPD mouse models to compare immune cell subsets in lung tissue and human peripheral blood (Figure 1A). Based on the gene signatures,^2^ we identified eleven major cell types, including CD4^+^ T cells (CD3D, CD4), CD8^+^ T cells (CD8A, CD8B), natural killer (NK) cells (NCAM1, KLRB1, NKG7), B cells (CD19, MS4A1), CD14^+^ monocytes (LYZ, CD14, CD68), CD16^+^ monocytes (LYZ, FCGR3A), conventional dendritic cells (cDCs; CLEC10A), plasmacytoid DCs (pDC; LILRA4),^3^ neutrophils (FCGR3B, CSF3R), progenitor cells (CD34) and megakaryocytes (PPBP) in the PBMCs (Figure 1B,C and Figure S1B,C). We observed a trend of decreased neutrophil abundance in BPD patients (Figure 1D), confirmed by FCM analysis of peripheral blood (Figure 1E and Figure S2A), which was also reflected in the BPD mouse model (Figure 1F and Figure S2B). To explore the causes of these immune differences, we investigated immune cell differentiation from hematopoietic stem cells (HSCs) in the bone marrow.^4^ FCM analysis of bone marrow samples from BPD mice revealed a significant increase in Lin^−^Sca1^+^cKit^+^ cells and long‐term HSCs in the BPD group (Figure S3). Enrichment analysis of gene expression from all PBMCs revealed oxidative phosphorylation as the primary pathway associated with upregulated genes in BPD (Figure 1G–I).

**FIGURE 1:**
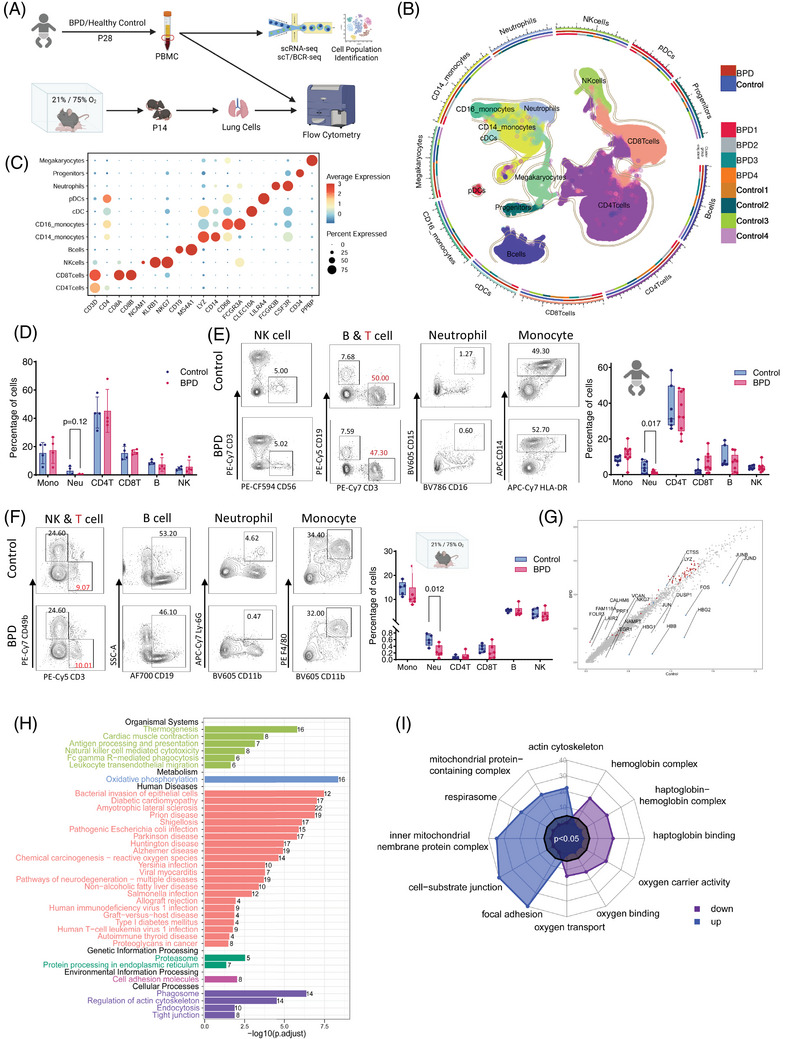
Single‐cell immune landscape of bronchopulmonary dysplasia (BPD) individuals. (A) Peripheral blood of preterm infants (GA<34 w) was collected after diagnosis with or without BPD for single‐cell RNA sequencing (scRNA‐seq) and flow cytometry (FCM). Mouse pups were exposed to either normal oxygen (21% O_2_) or high oxygen (75% O_2_) from the day of birth to postnatal 14 days (P14) and lungs were harvested. (B) UMAP plots of scRNA‐seq profiles showing the 11 distinct cell types. Monocytes and NK cells showed increased tendencies in the BPD group while B cells and neutrophils exhibited a decrease. (C) Dot plot representing the expression of canonical marker genes defining each peripheral blood mononuclear cell (PBMC) subset in scRNA‐seq profiles. (D) Percentage of each cell subset in scRNA‐seq data. No significant differences were observed between the control and BPD groups. (E) FCM analysis of nine BPD infants and seven non‐BPD healthy controls. Neutrophils were significantly reduced in the BPD group. (F) FCM analysis of BPD mice lungs. Neutrophils showed significant depletion in the BPD group, similar to BPD infants; *n* = 5–6/group. (G) Differentially expressed genes (DEGs) in PBMCs from BPD patients compared to healthy controls. Red (blue) indicates elevated (decreased) expression in BPD. (H) Kyoto Encyclopedia of Genes and Genomes (KEGG) pathway enrichment analysis of DEGs in PBMCs, highlighting significantly altered signalling and functional pathways between BPD patients and healthy controls. Bar colours represent different KEGG pathway categories: organismal systems (light green), metabolism (blue), human diseases (red), genetic information processing (dark green), environmental information processing (magenta) and cellular processes (purple). (I) Gene ontology (GO) enrichment analysis of the DEGs, showing significantly affected biological processes between BPD patients and healthy controls.

Using genes highly expressed in blood monocytes (S100A8, S100A9 and CSF3R) and classical markers (CD14, FCGR3A and LYZ),^3^ we classified monocytes into three subsets: CD14⁺ monocytes, CD16⁺ monocytes, and intermediate monocytes co‐expressing CD14 and FCGR3A. DCs were further divided into cDCs and pDCs. Mature neutrophils (FCGR3B, CCL4 and NAMPT) and granulocytes (CAMP, LCN2 and DEFA3)^5^ were identified (Figure 2A,B). In BPD patients, we observed a decrease in mature neutrophils and an increase in intermediate monocytes (Figure 2C), which are known to secrete pro‐inflammatory cytokines such as tumour necrosis factor (TNF)‐α, interleukin (IL)‐1β, and IL‐6 upon stimulation.^6^ FCM analysis confirmed the increased secretion of these cytokines by intermediate monocytes in BPD (Figure S4). Kyoto Encyclopedia of Genes and Genomes (KEGG) analysis unveiled that the differentially expressed genes (DEGs) in mature neutrophils were predominantly implicated in the oxidative phosphorylation pathway (Figure S5A and Figure 2D), consistent with the high‐energy demands of neutrophils in the injured area following mechanical ventilation.

**FIGURE 2:**
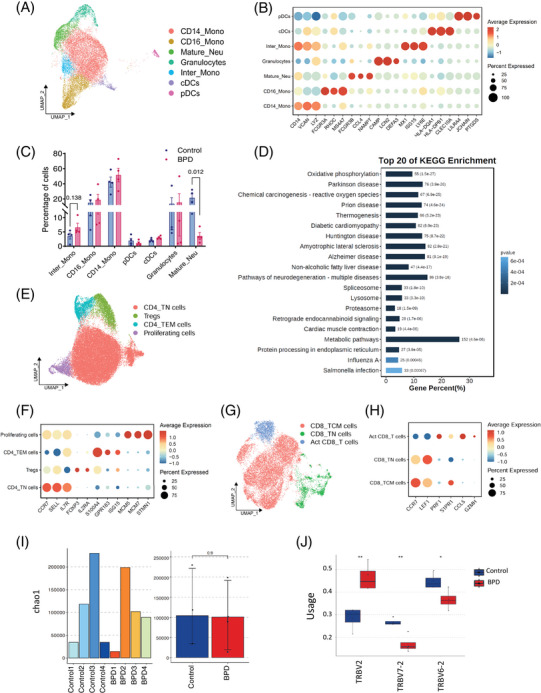
Characterization of myeloid and T cell subsets in bronchopulmonary dysplasia (BPD). (A) UMAP plots of myeloid cells, showing the seven distinct cell types identified. (B) Cluster markers of each myeloid cell type. (C) Proportions of each myeloid cell subsets in each human sample. (D) Bar plot showing the top 20 Kyoto Encyclopedia of Genes and Genomes (KEGG) pathways enriched in mature neutrophils from BPD patients compared to healthy controls. (E) UMAP plots of CD4^+^ T cells, showing the four distinct cell types that were identified. (F) Cluster markers of each CD4^+^ T subset.(G) UMAP plots of CD8^+^ T cells, showing the three distinct cell types were identified. (H) Cluster markers of each CD8^+^ T cell subset. (I) TCR repertoire diversity was estimated using the Chao1 Index. No significant difference was observed between healthy controls and BPD groups. (J) TRBV2, TRBV6‐2 and TRBV7‐2 showed significant changes in BPD among the beta chains. * *p* <.05;** *p* <.01; *n* = 4/group.

We identified seven T cell subsets. Within CD4⁺ T population, we categorized effector memory (S100A4, GPR183 and ISG15), naïve (CCR7, SELL and IL7R), regulatory T (Tregs; FOXP3 and IL2RA), and proliferating T cells (MCM5, MCM7 and STMN1)^7^ (Figure 2E,F). The distribution of CD4⁺ subsets was similar between BPD patients and healthy controls, with no significant differences (Figure S5B), suggesting immune dysregulation without major changes in CD4⁺ populations. Additionally, three CD8⁺ T cell subsets were identified: naïve (CCR7 and LEF1), central memory (PRF1 and S1PR1), and activated (CCL5 and GZMH)^8^ (Figure 2G,H). Central memory CD8⁺ T cells were slightly increased in BPD patients, indicating a potential role in infection control (Figure S5C). TCR clonal expansion analysis showed no significant differences in clonotypes or diversity between the two groups (Figure 2I and Figure S5D,E). However, changes in the distribution of V‐J pairs in β chains were observed, particularly in TRBV2, TRBV6‐2, and TRBV7‐2 in BPD (Figure 2J). In conclusion, specific TCR clonotypes in β chains may contribute to BPD pathogenesis.

Additionally, we identified three B cell subsets (Figure S6) and five NK cell subsets (Figure S7). CellChat analysis^9^ revealed a comprehensive interaction network involving all subsets (Figure S8A). Notably, BPD patients exhibited more cell‐to‐cell interactions compared to controls, with the strength of these interactions gradually increasing, potentially driven by elevated ligand and receptor expression during disease progression (Figure 3A,B and Figure S8B). We identified 22 ligand‐receptor pairs across 16 signalling pathways in BPD patients, with the GRN, CCL and B and T lymphocyte attenuator (BTLA) pathways unique to the BPD group (Figure 3C). B cells were central hubs in this network, particularly involved in BTLA signalling with T cells (Figure 3D and Figure S8C–E). The BTLA‐TNFRSF14 axis emerged as a major mediator of these interactions (Figure 3E). TNFRSF14 is a membrane‐bound receptor that activates nuclear factor‐kappa B, and BTLA‐TNFRSF14 is a well‐characterized ligand‐receptor pair.^10^ Treatment with an anti‐BTLA antibody in the BPD mouse model resulted in improved alveolar development and reduced inflammatory cytokines (Figure 3F,G). TNFRSF14 levels showed a parallel modulation (Figure 3H), suggesting that inhibiting the BTLA‐TNFRSF14 pathway may protect against lung damage and reduce inflammation in BPD.

**FIGURE 3:**
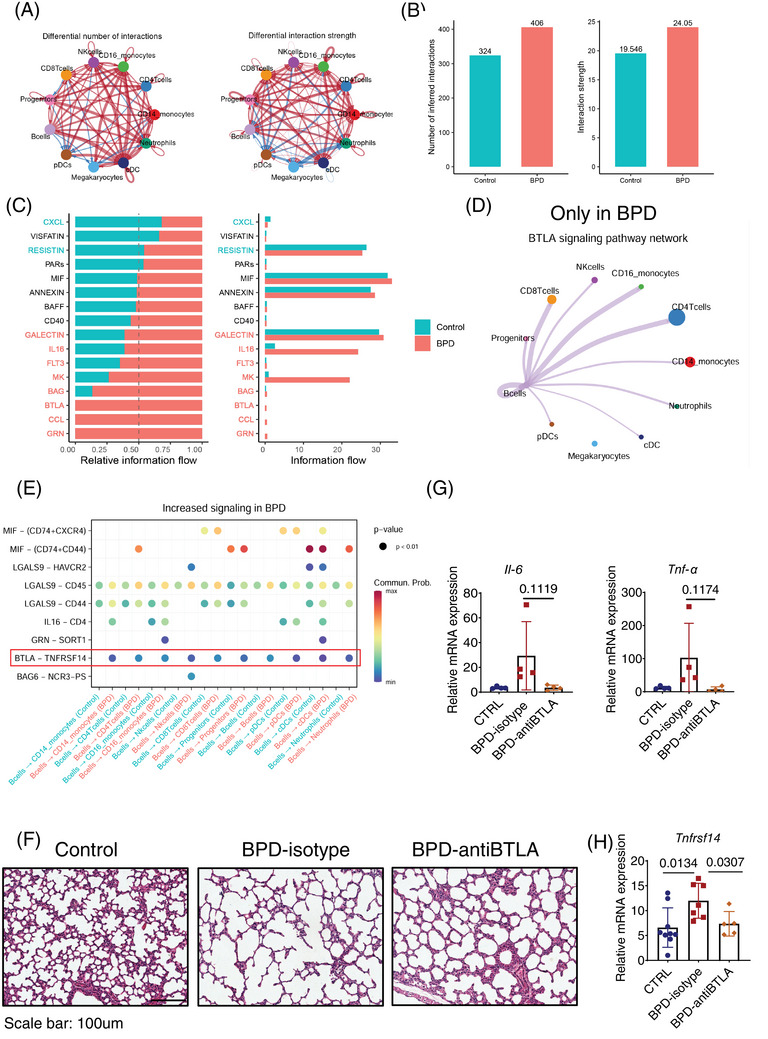
Cell‐to‐cell communication network inference analyzed by Cellchat. (A) Comparison of the number of significant ligand‐receptor pairs between pairs of cell populations, with red (blue) indicating increased (decreased) expression in bronchopulmonary dysplasia (BPD). (B) Bar plot displaying the number of inferred interactions and interaction strength between BPD and control group. (C) Bar plot showing differentially over‐expressed ligands and receptors for each group. (D) Inferred B and T lymphocyte attenuator (BTLA) signalling pathway networks in BPD single‐cell RNA sequencing (scRNA‐seq) profile. Circle sizes are proportional to the number of cells in each cell subset and edge width represents the communication probability. (E) Dot plot comparing the significant ligand‐receptor pairs between BPD and control groups. The highlighted BTLA‐TNFRSF14 signalling was up‐regulated in the BPD group. Dot color reflects communication probabilities and dot size represents computed *p*‐values. Empty space means the communication probability is zero. *p*‐Values are computed from a one‐sided permutation test. (F) Representative HE‐stained photomicrographs of the lung tissues from control or BPD mice treated with vehicle or anti‐BTLA antibody; *n* = 5–6/group. Scale bar: 100 µm. (G) Relative mRNA expression of Il‐6 and Tnf‐α in lung tissues from control or BPD‐induced mice treated with vehicle or anti‐BTLA antibody. (H) Relative mRNA expression of Tnfrsf14 in lung tissues from control or BPD‐induced mice treated with vehicle or anti‐BTLA antibody.

In conclusion, we found significant alterations in both myeloid and lymphoid subsets, with neutrophils undergoing metabolic reprogramming toward oxidative phosphorylation. Progenitor cell analysis in BPD mouse models revealed specific alterations in HSCs. The BTLA‐TNFRSF14 signalling axis plays a key role in BPD pathogenesis, and blocking BTLA in mouse models reduces disease severity, suggesting potential therapeutic effects.

## AUTHOR CONTRIBUTIONS

Yufeng Liu, Wangkai Liu, Yushan Li and Chun Yan designed experiments. Yufeng Liu, Wangkai Liu, Yushan Li, Chun Yan, Xiaoyu Lin, Qiong Meng, Sitao Li and Limei Zhong performed the experiments. Yufeng Liu, Wangkai Liu, Yushan Li, Chun Yan and Ruoxing Zhou analyzed data. Yufeng Liu, Wangkai Liu, Yushan Li, Yanfang Tan and Chun Yan wrote the manuscript.

## CONFLICT OF INTEREST STATEMENT

The authors declare no conflict of interest.

## FUNDING INFORMATION

This work was supported by grants from National Natural Science Funds (No. 82171695); Science and Technology Program of Guangzhou (SL2024A03J01319; SL2024A04J00240); Natural Science Foundation of Guangdong Province, China (2022A1515010031); Basic and Applied Basic Research Fund of Guangdong Province (2022A1515012548).

## ETHICS STATEMENT

This study was approved by the Institutional Ethics Committee for Clinical Research and Animal Trials of the First Affiliated Hospital of Sun Yat‐sen University (No. [2020]521).

## Supporting information



Supporting Information

Supporting Information

Supporting Information

Supporting Information

Supporting Information

Supporting Information

Supporting Information

Supporting Information

Supporting Information

## Data Availability

The raw sequence data have been deposited in the Genome Sequence Archive in the National Genomics Data Center, China National Center for Bioinformation/Beijing Institute of Genomics, Chinese Academy of Sciences (GSA‐Human: HRA007764). The remaining data are available from the authors upon request.
