# Supplementary figures and images for: Single‐cell RNA sequencing of peripheral blood mononuclear cells from bronchopulmonary dysplasia
Source: Clin Transl Med. 2025 Mar 17;15(3):e70276. doi: 10.1002/ctm2.70276 (PMC11913593; doi:10.1002/ctm2.70276)

A

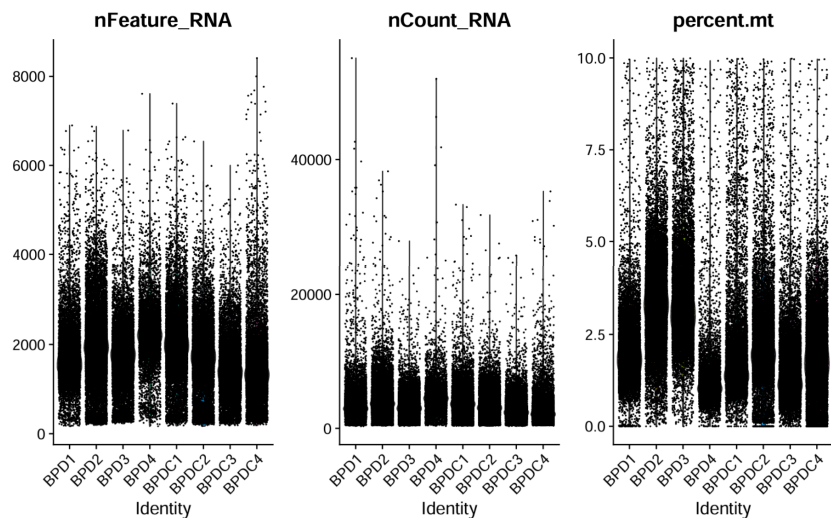

B

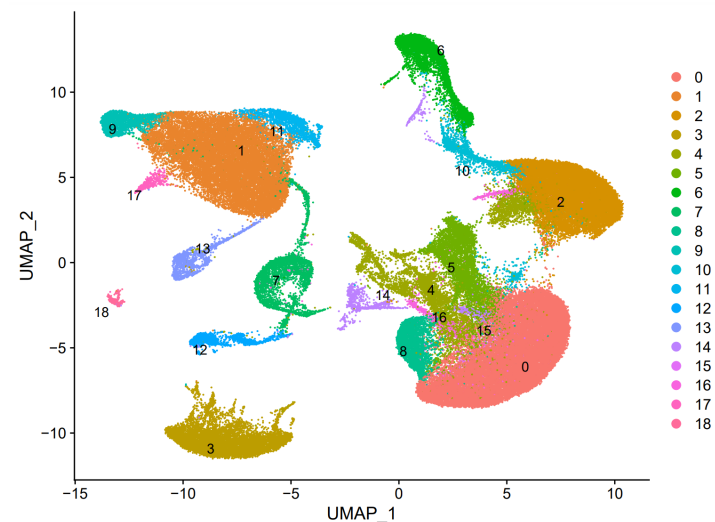

C

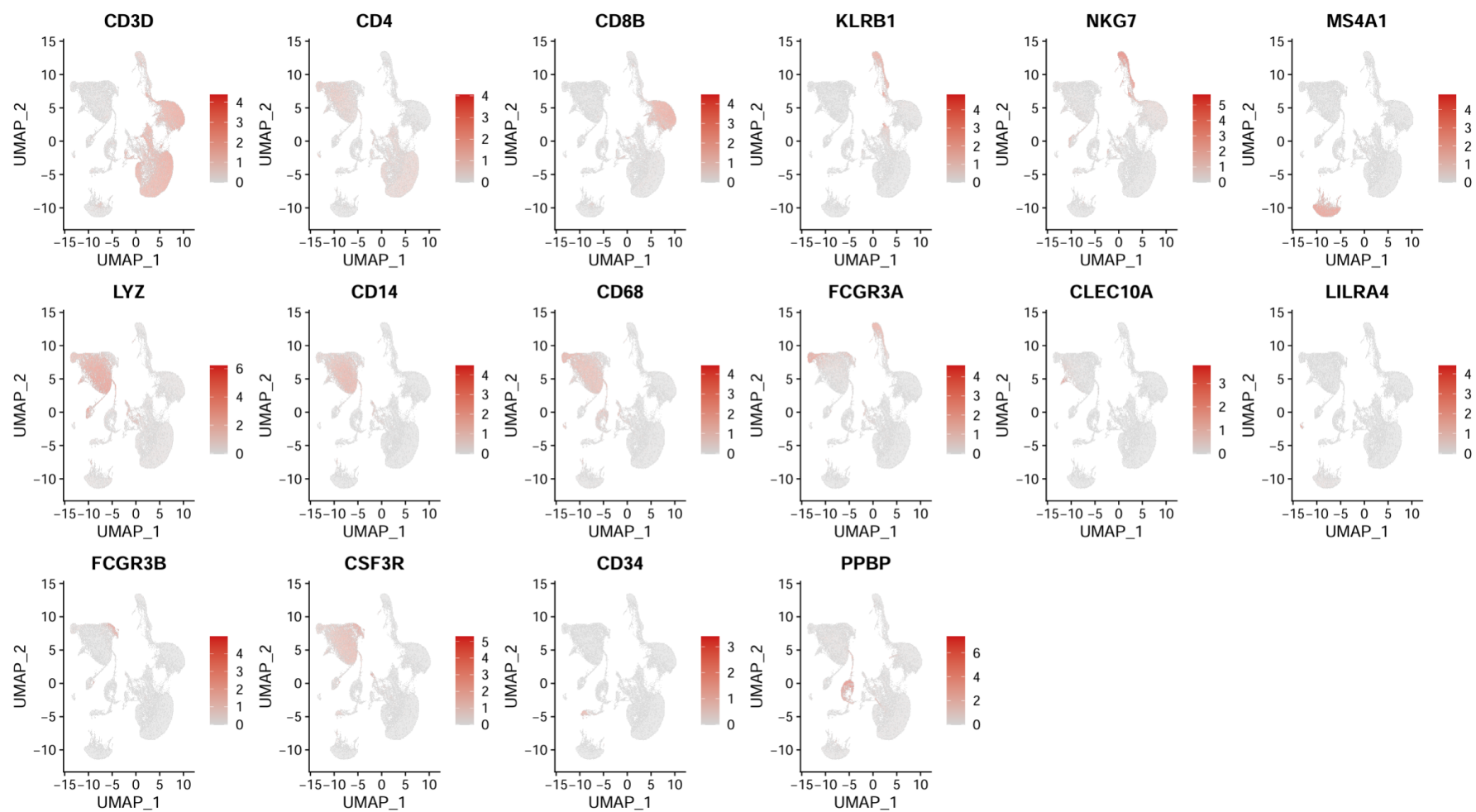

Supplement: Supplementary file 1 — Supporting Information [file CTM2-15-e70276-s001.pdf]

A

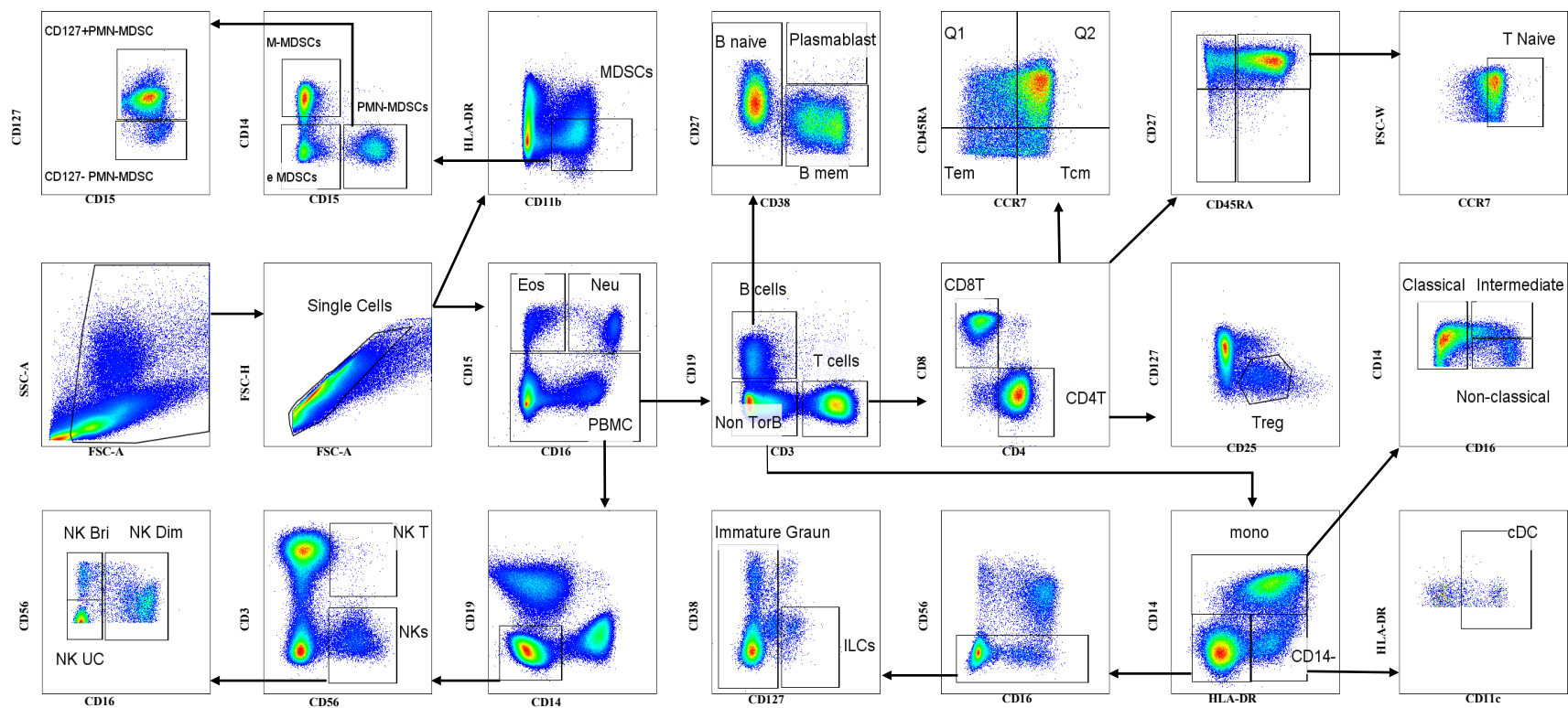

B

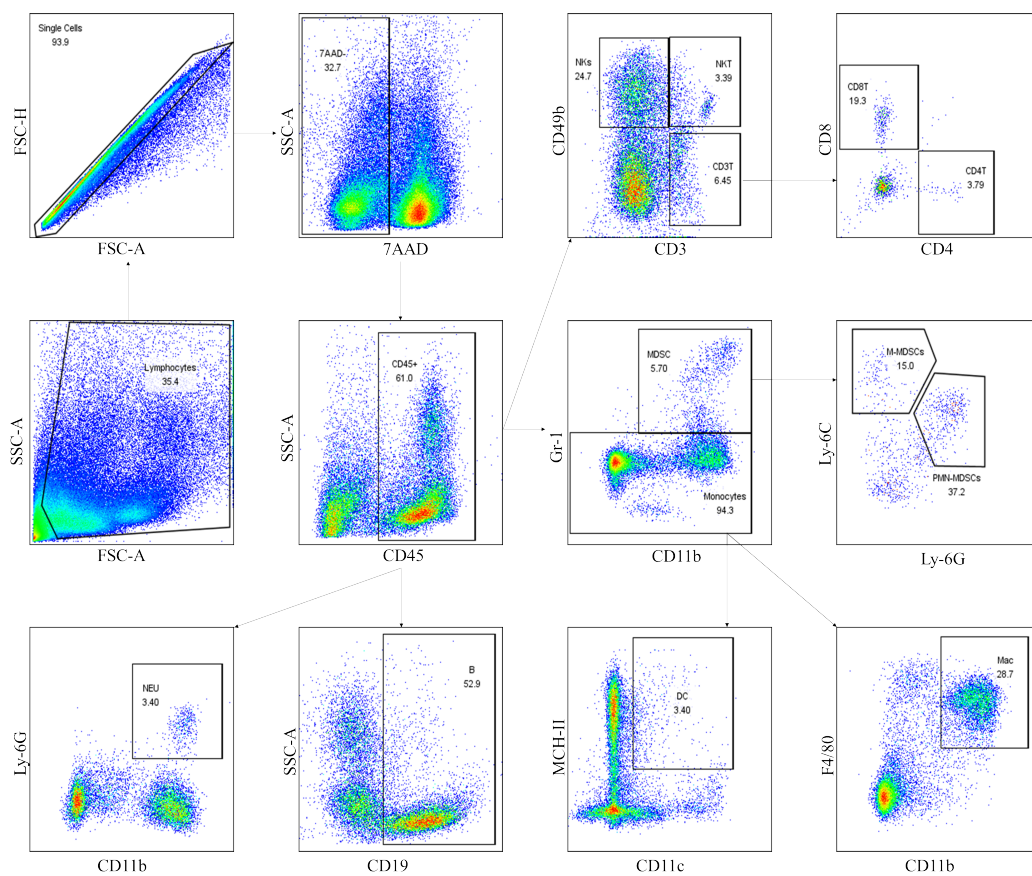

Supplement: Supplementary file 3 — Supporting Information [file CTM2-15-e70276-s002.pdf]

A

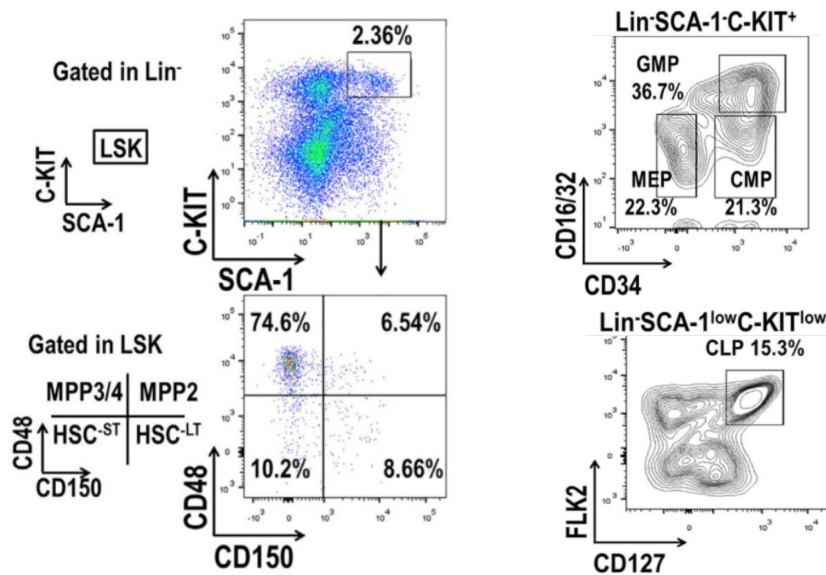

B

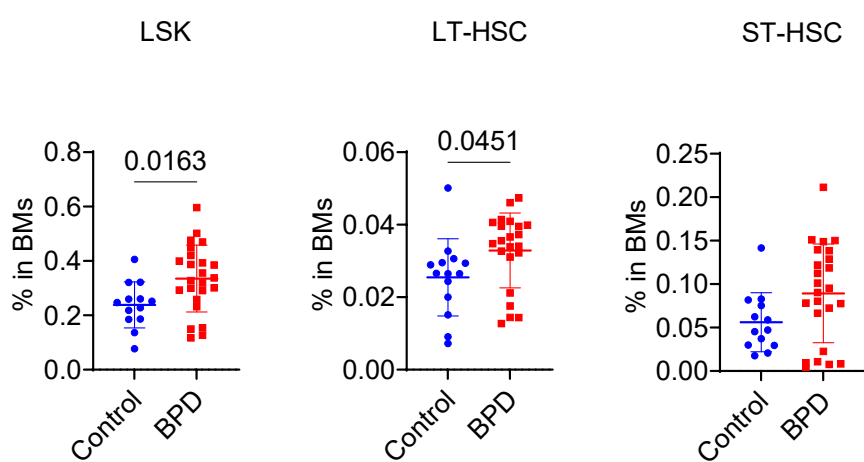

C

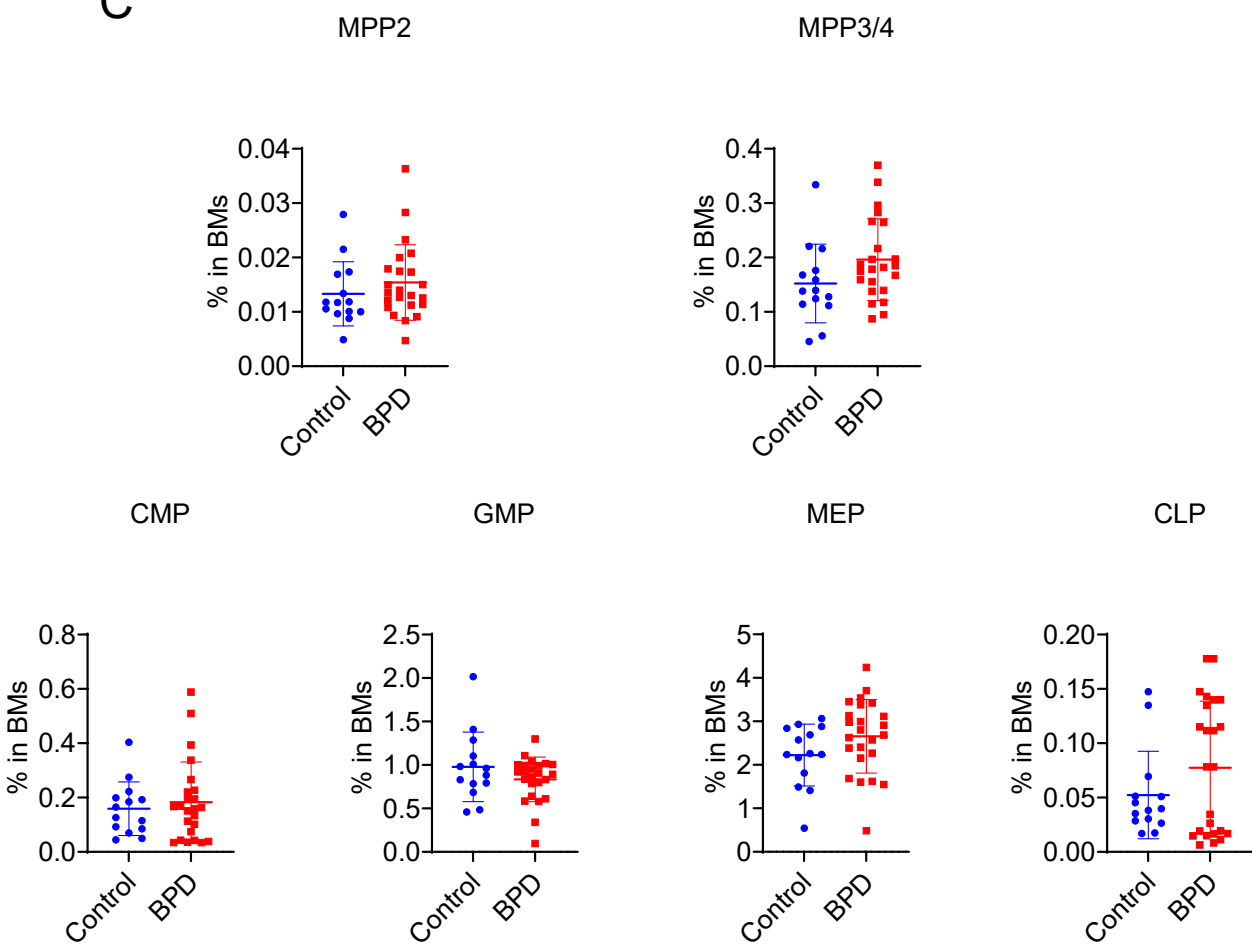

Supplement: Supplementary file 4 — Supporting Information [file CTM2-15-e70276-s007.pdf]

A

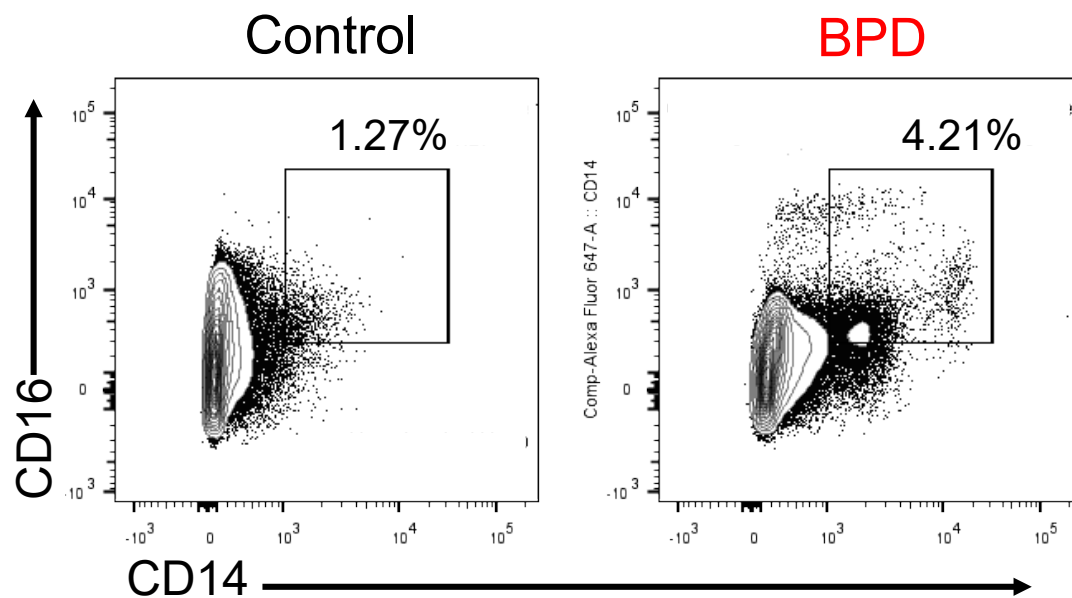

B

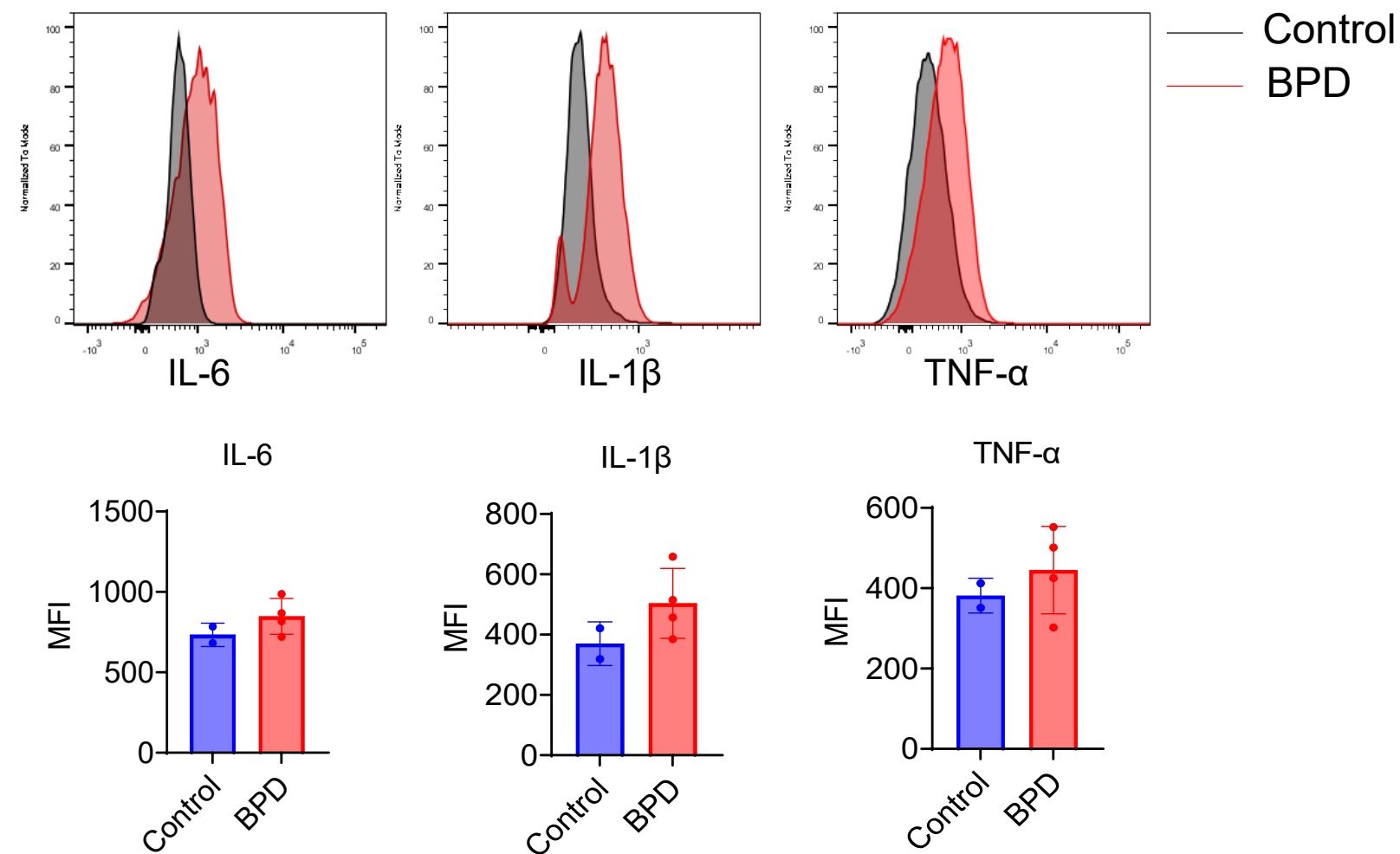

Supplement: Supplementary file 5 — Supporting Information [file CTM2-15-e70276-s003.pdf]

[illegible]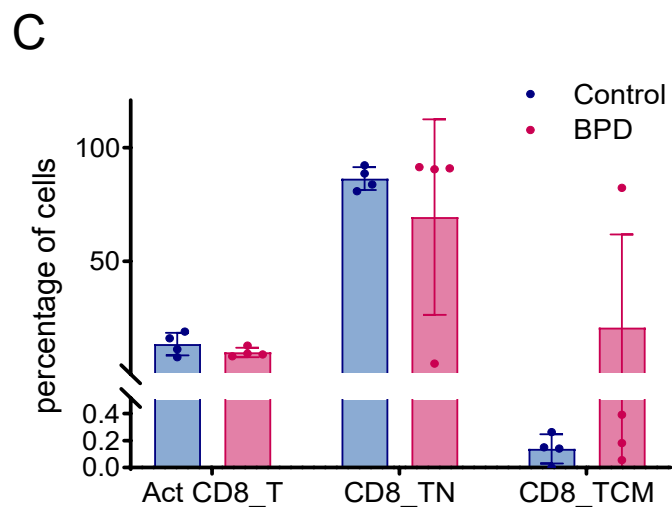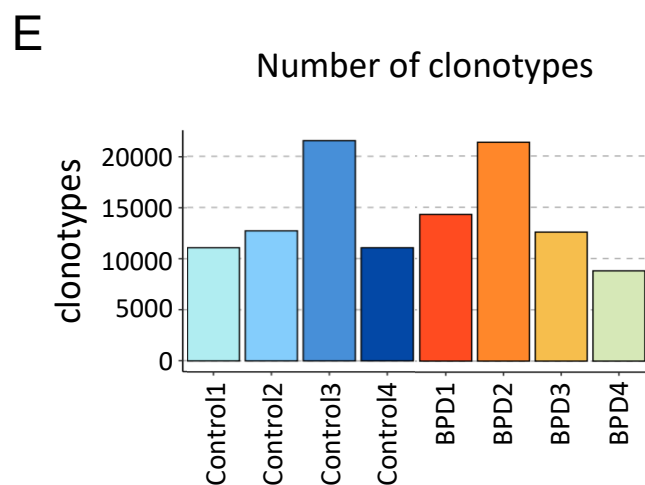

A

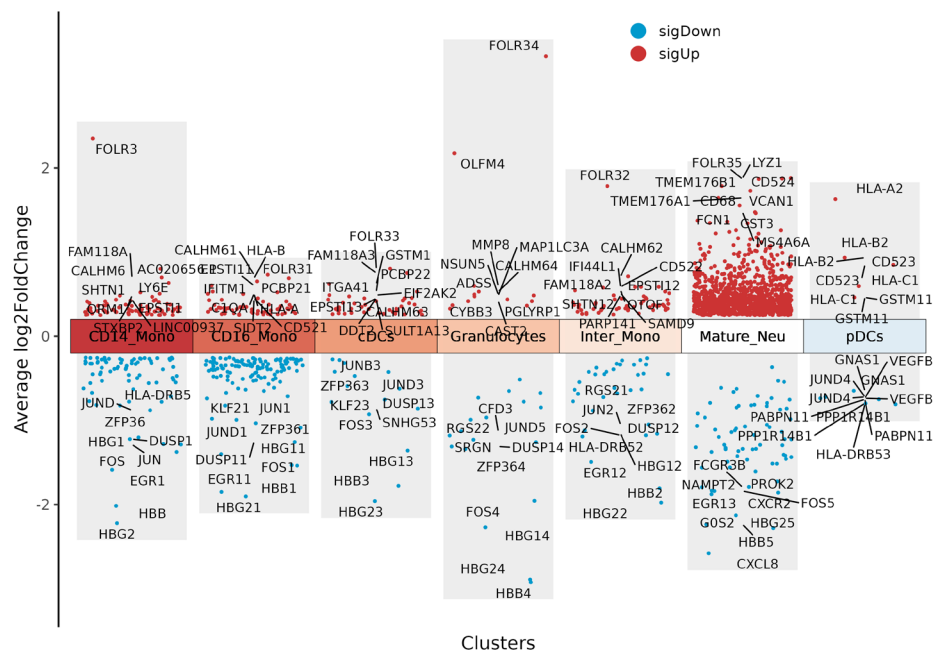

B

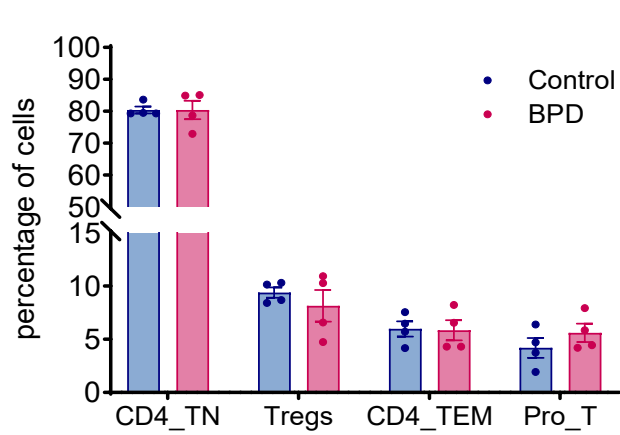

C

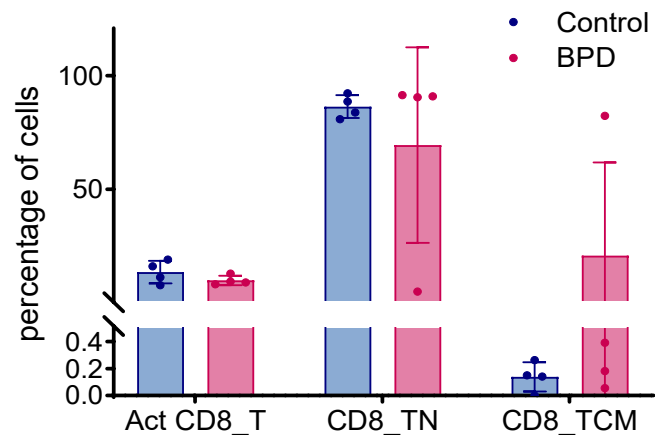

D

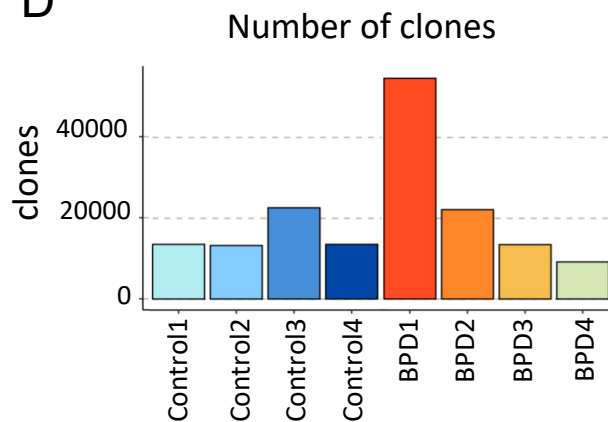

E

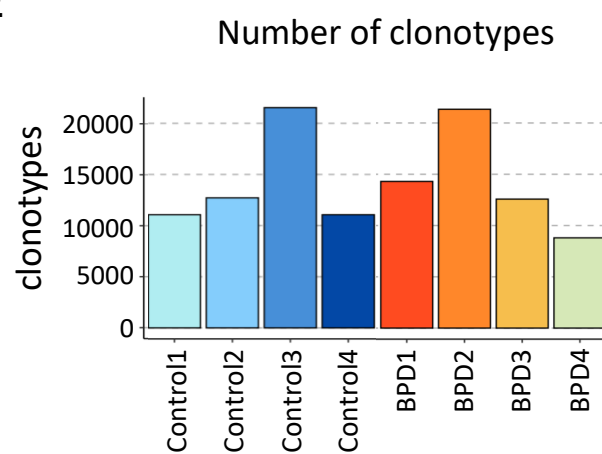

Supplement: Supplementary file 6 — Supporting Information [file CTM2-15-e70276-s005.pdf]

A

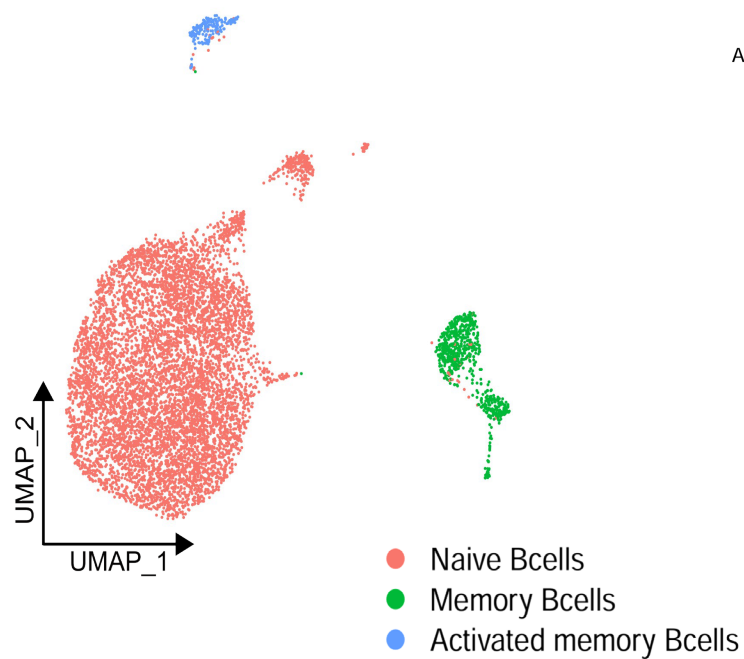

B

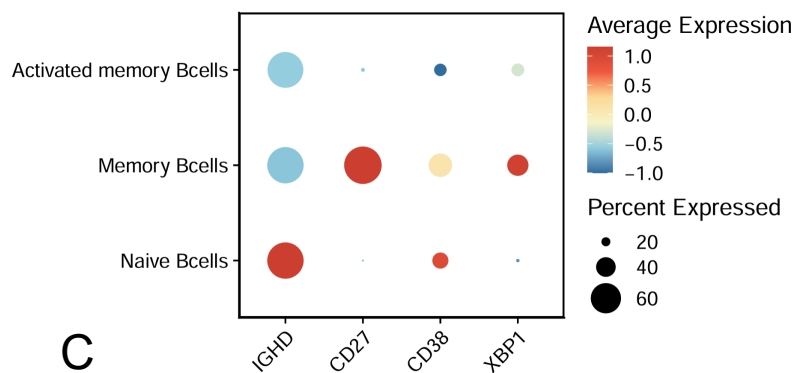

C

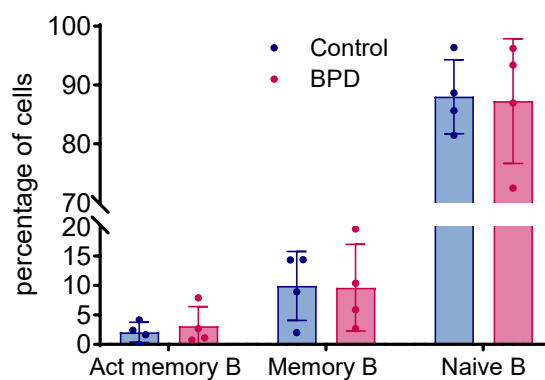

D

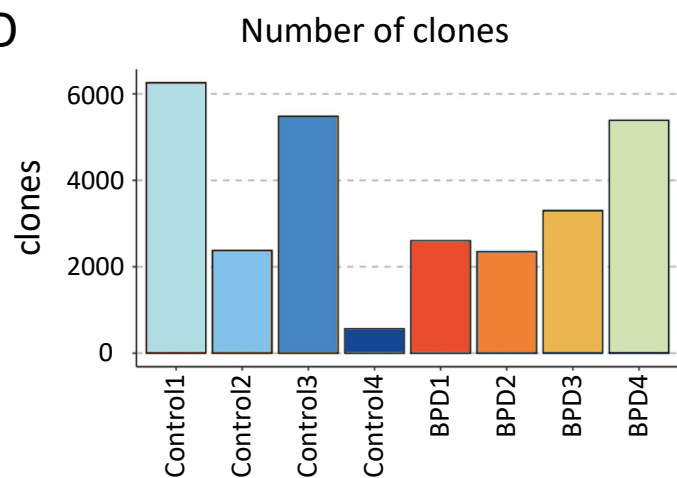

E

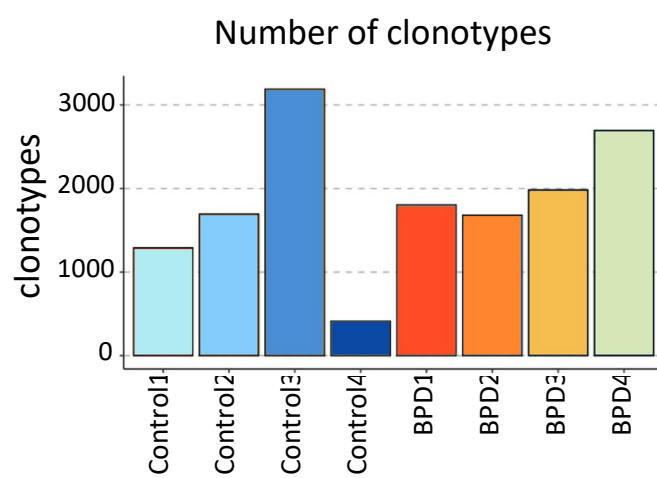

F

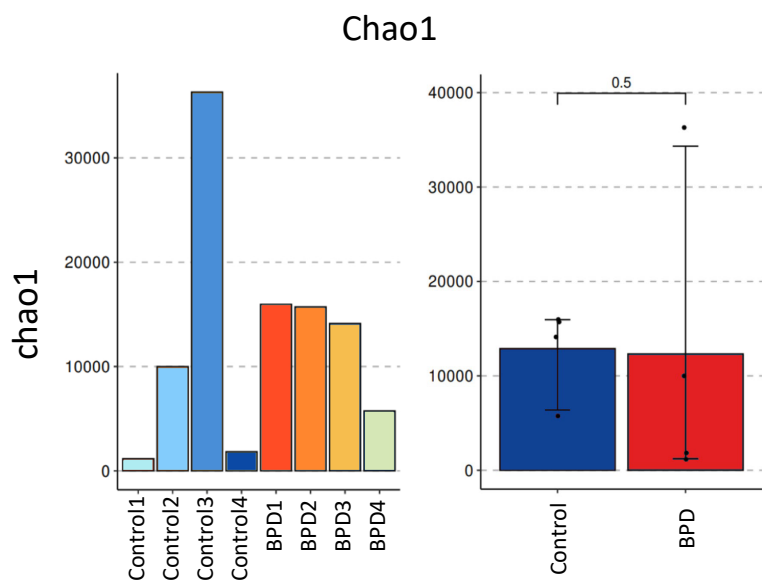

G

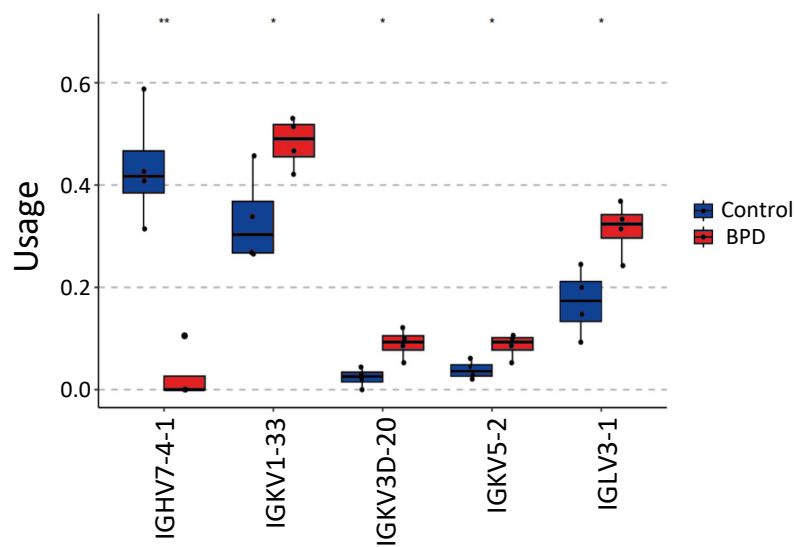

Supplement: Supplementary file 7 — Supporting Information [file CTM2-15-e70276-s006.pdf]
