# Supplementary material for: Single‐cell RNA sequencing of peripheral blood mononuclear cells from bronchopulmonary dysplasia
Source: Clin Transl Med. 2025 Mar 17;15(3):e70276. doi: 10.1002/ctm2.70276 (PMC11913593; doi:10.1002/ctm2.70276)
Supplement: Supplementary file 2 — Supporting Information [file CTM2-15-e70276-s008.docx]

Supplementary Information for

**Single-cell RNA sequencing of peripheral blood mononuclear cells from** **bronchopulmonary dysplasia**

**This PDF file includes:** Materials and methods; Figure legends; Supplementary Tables

# Materials and methods

## Participants

The study sample consisted of 11 BPD patients (2 female) and 9 non-BPD preterm infants (2 female). All infants were recruited from the First Affiliated Hospital of Sun Yat-sen University between December 2021 and November 2022, with approval from the Institutional Ethics Committee for Clinical Research and Animal Trials of the hospital (No. [2020]521). BPD was diagnosed according to the criteria established by the National Institute of Child Health and Human Development (NICHD) in 2001 [1]. Infants with infections, inflammation, congenital heart disease, congenital malformations, chromosomal abnormalities, or anemia were excluded. The indicators assessed included C-reactive protein (CRP), procalcitonin (PCT), white blood cell count (WBC), neutrophils (NEUT), and platelet count (PLT). At the time of sampling, all indicators were within the normal range for the corresponding postnatal age. Blood samples were obtained between 28 and 35 days after birth. Four infants (36.4%) in the BPD group experienced early-onset sepsis. All BPD infants (100%) required respiratory support at the time of sampling, with the severity distribution as follows: 7 severe, 2 moderate, and 2 mild cases. Detailed characteristics of the human subjects for single-cell RNA sequencing (scRNAseq) and flow cytometry (FCM) analyses are presented in Supplementary Tables 1 and 2.

## Animals

C57BL/6 mice were obtained from the Laboratory Animal Center of Sun Yat-sen University. Animal experiments were approved by Guangdong HUA WEI Testing Co., Ltd. Pregnant C57BL/6 mice were housed individually in cages and provided with ad libitum food. Pups born on the same day were randomly assigned to either the hyperoxia or normoxia group, with 7 pups per group on the day of birth (P0). The cages were subsequently exposed to either room air (21% O_2_) or hyperoxia (75% O_2_) until the harvest day (P14). The hyperoxia cage was maintained in a sealed PMMA box with continuous oxygen concentration monitoring. To minimize oxygen toxicity to the dams and reduce pup mortality, the hyperoxia groups were returned to normoxia for 3 hours daily . All mice were kept in a specific pathogen-free (SPF) environment and maintained on a 12-hour light/dark cycle.

## Preparation of single-cell suspensions

For human participants, blood was collected in BD Vacutainer (EDTA) tubes, diluted with phosphate-buffered saline (PBS) to twice its original volume, and layered over 3 mL of Ficoll per 2 mL of diluted blood in 15 mL tubes. The samples were centrifuged at 600 × g for 30 minutes at room temperature (RT), and the peripheral blood mononuclear cell (PBMC) layer was isolated. This layer was transferred to a fresh conical tube, diluted again with PBS to twice its volume, and subjected to further centrifugation. The supernatant was discarded, and the PBMC pellet was gently resuspended in 5 mL of red blood cell lysis buffer and incubated for 5 minutes at RT. The PBMCs were then resuspended in 2 mL FACS buffer, and a 5 µL aliquot was diluted 1:10 (v/v) for cell counting. Subsequently, FCM analysis and scRNA-seq analysis were performed.

For animals, pups were euthanized at P14 using isoflurane anesthesia. The lungs were then excised and sectioned into 1 mm^3^ fragments. The minced lung tissue was gently homogenized through a 70 μm sterile cell strainer. The resulting cell suspension was centrifuged at 2000 rpm for 5 minutes at 4°C. Red blood cells were lysed using a red blood cell lysis buffer. For bone marrow samples, the right lower limb of the mouse was dissected, the femur and tibia were separated, and the epiphysis was cut. Bone marrow was rinsed out by injecting 15 mL PBS solution with a 1 mL syringe. After centrifugation, red blood cells were lysed, and the final concentration was adjusted to 1 × 10^6^ cells per mL.

## Flow cytometry analysis

Single-cell suspensions (1 × 10^6 cells/mL) were stained with human or mouse-specific antibody cocktails for 30 minutes at 4°C. After washing with FACS buffer, the cells were analyzed using a BD LSRFortessa™ Cell Analyzer, and data were processed with FlowJo (v10.8.1). Antibody details are provided in Supplementary Tables 3-4.

## Single-cell RNA sequencing

Sorted PBMCs were resuspended in PBS containing 0.04% bovine serum albumin and loaded into Chromium microfluidic chips with 5’ chemistry for barcoding using a 10× Chromium Controller (10× Genomics). RNA was extracted from the barcoded cells, reverse transcribed, and sequencing libraries were prepared using the Chromium Single Cell 5’ v2 Reagent Kit (10× Genomics), following the manufacturer's protocol. Sequencing was conducted on an Illumina NovaSeq 4000 platform, as per the manufacturer’s instructions (Illumina).

## Single-cell data integration and analysis

The Cell Ranger (v.4.0.0) package was used to align reads to the GRCh38 genome, filter barcodes, and calculate unique molecular identifiers (UMIs). Raw data were processed with the Seurat (v4.3.0) package in R. Low-quality cells (fewer than 300 genes or >10% mitochondrial content) were excluded (Supplementary Fig. 1A), and doublets were removed using the DoubletFinder (v2.0.3) package. Data were normalized, and the top 2000 variable genes were identified for principal component analysis (PCA). The principal components were used for clustering, and distinct cell clusters were annotated. Finally, UMAP algorithm was applied for dimensionality reduction.

## Identification of cell marker genes

Cell cluster-specific marker genes were identified using Seurat's “FindAllMarkers” function. Differentially expressed genes (DEGs) were determined by comparing each cluster to all others using the Wilcoxon rank-sum test. DEGs with Bonferroni-corrected p-values < 0.01 and an average expression > 1.5-fold higher in the cluster than in other clusters were considered significant. Canonical markers and top-ranked DEGs were used to annotate cell types for each cluster.

## Functional analysis

The “limma” (v3.56.2) package was used to identify DEGs between clusters, with significant genes selected based on false discovery rate (FDR) < 0.05 and |log2FC| > 2. Pathway and functional enrichment analysis were conducted using KEGG and GO databases with R packages: “ClusterProfiler” (v4.8.3), “org.Hs.eg.db” (v3.17.0), “ggplot2” (v3.4.3), and “enrichplot” (v1.20.1) [2].

## CellChat analysis

Ligand-receptor interactions between cell clusters were analyzed using the “CellChat” (v1.1.2) R package[3], which provides extensive databases of ligands, receptors, cofactors, and their interactions. This toolkit facilitates the identification of novel intercellular communication pathways and the creation of communication atlases. Ligand and receptor expression levels were normalized to total reads across coding genes, and average expression values were calculated for each single-cell cluster to evaluate intercellular interactions.

## TCR and BCR repertoire analyses

TCR and BCR sequences from T and B cells were extracted using the Cell Ranger V(D)J pipeline with GRCh38 as the reference genome. Clonotypes were assigned based on unique TRA-TRB (TCR) and IGH-IGK/IGL (BCR) pairs, with clonotypes defined as clonal if present in at least two cells. The immunarch package was used for clonotype analysis. V(D)J sequence assembly and clonotype calling were performed with a reference specific to each sample. The usage of TRAV/J, TRBV/J, IGHV/J, and IGLV/J gene segments was analyzed and compared, and the distribution of CDR3 amino acid (aa) lengths was assessed by counting segments with identical lengths.

## Neutralization of BTLA

Hyperoxia-exposed mice were subjected to intraperitoneal injections of anti-BTLA antibody (6μg/g; clone 6A6; BioLegend). Control mice received corresponding isotype control injections. Injections were initiated on P3 and administered every other day until the end of the experiment.

## Histological Tests

Mice were euthanized via an overdose of isoflurane. Lung tissues were subsequently embedded in paraffin, followed by deparaffinization and dehydration. Hematoxylin and eosin staining was applied to the tissue sections.

## Quantitative Reverse Transcription PCR (RT‒qPCR)

Total RNA was extracted from lung tissues using the RNA Extraction Kit (RN001, ESScience). The isolated RNA was then used for cDNA synthesis with the RNA Reverse Transcription Kit (RR036A, Takara). The resulting cDNA was amplified using the SYBR Green qPCR SuperMix (AQ608, TRANS), and real-time detection of fluorescent signals was performed. Gene expression was quantified using the 2^−ΔΔCt^ method, with β-actin as the internal reference. Primer sequences are listed in Supplementary Table 5.

## Statistical analysis

For FCM data, statistical analyses were performed using GraphPad Prism 8. Normally distributed variables were compared between two groups using two-tailed unpaired t-tests, while non-normally distributed variables were analyzed using two-tailed Wilcoxon rank-sum tests. Data are presented as mean ± standard deviation (SD). scRNA-seq data analyses were conducted in R (v4.2.1), with DEGs identified using the Wilcoxon test within the FindMarkers function of Seurat. TCR/BCR V(D)J gene usage bias was assessed using a combination of the FDR test and Fisher's exact test. A p-value < 0.05 was considered significant, with significance levels indicated by asterisks: *p < 0.05.

**Reference:**

1. Jobe AH, Bancalari E (2001) Bronchopulmonary dysplasia. Am J Respir Crit Care Med 163:1723–1729. https://doi.org/10.1164/ajrccm.163.7.2011060

2. Yu G, Wang L-G, Han Y, He Q-Y (2012) clusterProfiler: an R package for comparing biological themes among gene clusters. OMICS 16:284–287. https://doi.org/10.1089/omi.2011.0118

3. Jin S, Guerrero-Juarez CF, Zhang L, et al (2021) Inference and analysis of cell-cell communication using CellChat. Nat Commun 12:1088. https://doi.org/10.1038/s41467-021-21246-9

# Figures legend

**Figure 1 Single Cell Immune Landscape of BPD Individuals.** (A) Peripheral blood of preterm infants(GA＜34w) was collected after diagnosis with or without BPD for scRNA-seq and FCM. Mouse pups were exposed to either normal oxygen (21% O_2_) or high oxygen (75% O_2_) from the day of birth to postnatal 14 days (P14) and lungs were harvested. (B) UMAP plots of scRNA-seq profiles showing the 11 distinct cell types. Monocytes and NK cells showed increased tendencies in BPD group while B cells and neutrophils exhibited a decrease. (C) Dot plot representing the expression of canonical marker genes defining each PBMC subset in scRNA-seq profiles. (D) Percentage of each cell subset in scRNA-seq data. No significant differences were observed between the control and BPD groups. (E) FCM analysis of 9 BPD infants and 7 non-BPD healthy controls. Neutrophils were significantly reduced in the BPD group. (F) FCM analysis of BPD mice lungs. Neutrophils showed significant depletion in the BPD group, similar to BPD infants; n = 5-6/group. (G) DEGs in PBMCs from BPD patients compared to healthy controls. Red (blue) indicates elevated (decreased) expression in BPD. (H) KEGG pathway enrichment analysis of DEGs in PBMCs, highlighting significantly altered signaling and functional pathways between BPD patients and healthy controls. Bar colors represent different KEGG pathway categories: Organismal Systems (light green), Metabolism (blue), Human Diseases (red), Genetic Information Processing (dark green), Environmental Information Processing (magenta), and Cellular Processes (purple). (I) GO enrichment analysis of the DEGs, showing significantly affected biological processes between BPD patients and healthy controls.

**Figure 2 Characterization of Myeloid and T Cell Subsets in BPD.** (A) UMAP plots of myeloid cells, showing the 7 distinct cell types identified. (B) Cluster markers of each myeloid cell type. (C) Proportions of each myeloid cell subsets in each human sample. (D) Bar plot showing the top 20 KEGG pathways enriched in mature neutrophils from BPD patients compared to healthy controls. (E) UMAP plots of CD4^+^ T cells, showing the 4 distinct cell types that were identified. (F) UMAP plots of CD8^+^ T cells, showing the 3 distinct cell types that were identified. (G) Cluster markers of each CD4^+^ T subset. (H) Cluster markers of each CD8^+^ T cell subsets. (I) TCR repertoire diversity estimated using the Chao1 Index. No significant difference was observed between healthy controls and BPD groups. (J) TRBV2, TRBV6-2, and TRBV7-2 showed significant changes in BPD among the beta chains. * p＜ 0.05;** p＜ 0.01; n=4/group.

**Figure 3 Cell-to-Cell Communication Network Inference Analyzed by Cellchat.** (A) Comparison of the number of significant ligand-receptor pairs between pairs of cell populations, with red (blue) indicating increased (decreased) expression in BPD. (B) Bar plot displaying the number of inferred interactions and interaction strength between BPD and control group. (C) Bar plot showing differentially over-expressed ligands and receptors for each group. (D) Inferred BTLA signaling pathway networks in BPD scRNA-seq profile. Circle sizes are proportional to the number of cells in each cell subset and edge width represents the communication probability. (E) Dot plot comparing the signiﬁcant ligand-receptor pairs between BPD and control groups. The highlighted BTLA-TNFRSF14 signaling was up-regulated in BPD group. Dot color reﬂects communication probabilities and dot size represents computed p-values. Empty space means the communication probability is zero. P-values are computed from one-sided permutation test. (F) Representative HE-stained photomicrographs of the lung tissues from control or BPD mice treated with vehicle or anti-BTLA antibody; n = 5-6/group. Scale bar: 100 μm. (G) Relative mRNA expression of Il-6 and Tnf-α in lung tissues from control or BPD-induced mice treated with vehicle or anti-BTLA antibody. (H) Relative mRNA expression of Tnfrsf14 in lung tissues from control or BPD-induced mice treated with vehicle or anti-BTLA antibody.

**Supplementary Figure 1 Characteristics of PBMC scRNA-seq data.** **(A)** Violin plots depicting the distribution of feature counts, RNA counts, and mitochondrial percentages of each scRNA-seq sample post-filtering. **(B)** UMAP plot illustrating the distribution of PBMCs. Each point represents one cell, with coloring based on the sample origin. **(C)** UMAP plot showing the expression of canonical markers across main cell lineages by color. Each point represents one cell.

**Supplementary Figure 2 Gating strategy of mFCM data.** **(A)** Flow loop gate strategy of human peripheral blood. **(B)** Flow loop gate strategy of BPD mice lung tissue.

**Supplementary Figure 3 The flow cytometry analysis of cells in bone marrow.** **(A)** Representative ﬂow cytometry gating plot of MPP2, MPP3/4, LSK, HT-HSC, and ST-HSC. **(B)** Proportions of LSK, HT-HSC, and ST-HSC in mouse bone marrow (n=13/22). **(C)** Proportions of MPP2, MPP3/4, CMP, GMP, MEP, and CLP in mouse bone marrow (n=13/22). LSK, Lin-Sca1+cKit+; HT-HSC, Hematopoietic Stem Cell (long-term); ST-HSC, Hematopoietic Stem Cell (short-term); MPP2, Multipotent Progenitor 2; MPP3/4, Multipotent Progenitor 3/4; CMP, Common Myeloid Progenitor; GMP, Granulocyte-Macrophage Progenitor; MEP, Megakaryocyte-Erythroid Progenitor; CLP, Common Lymphoid Progenitor.

**Supplementary Figure 4 Increased expression of inflammatory cytokines in peripheral intermediate monocytes from BPD patients. (A)** Representative ﬂow cytometry gating plot of intermediate monocytes. **(B)** MFI of IL-6, IL-1β, and TNF-α in intermediate monocytes from control and BPD patients. MFI: mean fluorescence intensity.

**Supplementary Figure 5 Related to Figure 2.** (A) Combined dot plot representing the DEGs between BPD and control individuals across all myeloid cell subsets in scRNA-seq profiles, with red (blue) color indicating elevated (decreased) expression in BPD. (B) The respective percentages of each distinct CD4^+^ T cell type in control group and BPD group. (C) The respective percentages of each distinct CD8^+^ T cell type in control group and BPD group. (D) The number of clones in TCR. Both the quantity and proportion of T cell clonotypes increased in BPD patients. (E) The number of clonotypes in TCR. Both the quantity and proportion of T cell clonotypes increased in BPD patients.

**Supplementary Figure 6 Characterization of B cells and BCRs.** (A) UMAP plots of B cells, showing the 3 distinct cell types that were identified and their respective percentages in control group and BPD group. (B) The cluster markers of each B cell subsets. (C) The respective percentages of each distinct B cell type in control group and BPD group. (D) The number of clones and clonotypes in BCR. Both the quantity and proportion of T cell clonotypes increased in BPD patients. (E) The diversity of BCR repertoire were estimated using Chao1 Index. There was no significant difference between control group and BPD group. (F) Chao1 index showed no significant difference in the diversity of BCR repertoire. (G) Histogram showed genes IGHV7-4-1, IGKV1-33, IGK3D-20, IGKV5-2, and IGKV3-1 were changed significantly between the two groups. * p＜ 0.05;** p＜ 0.01; n=4/group.

**Supplementary Figure 7 NK Cell Heterogeneity in BPD. (A)** UMAP plots of NK cells, showing the 5 distinct cell types that were identified. **(B)** Dot plots showing the cluster markers of each NK cell subset. **(C)** The percentages of each NK cell subset, showing that NKT were decreased significantly in BPD group. n=4/group. **(D)** KEGG analysis of NKT-related genes suggested that adherens junction pathways, Wnt signaling pathway, and Hippo signaling pathway were enriched. The label color highlights different pathway categories or gene number information: Human Diseases (red), Organismal Systems (cyan), Cellular Processes (blue), Environmental Information Processing (yellow), gene number of corresponding pathway (purple), DEGs in the corresponding pathway (dark green), other DEGs (light green).

**Supplementary Figure 8 Additional details on Cellchat analysis, related to Fig. 6.** **(A)** Number of significant ligand-receptor pairs between any pair of two cell populations. The edge width is proportional to the indicated number of ligand-receptor pairs. **(B)** Heatmap shows the relative importance of each cell group based on the computed network centrality. **(C)** Inferred GRN signaling pathway networks in BPD scRNA-seq profile. Circle sizes are proportional to the number of cells in each cell subset and edge width represents the communication probability. **(D)** Inferred CCL signaling pathway networks in BPD scRNA-seq profile. Circle sizes are proportional to the number of cells in each cell subset and edge width represents the communication probability. **(E)** Dot plot comparing the signiﬁcant ligand-receptor pairs between BPD and control groups. The highlighted GRN-SORT1 and CCL5-CCR1 signaling were up-regulated in BPD group. Dot color reﬂects communication probabilities and dot size represents computed p-values. Empty space means the communication probability is zero. P-values are computed from one-sided permutation test.

# Supplementary Tables

## Supplementary Table 1 scRNA-seq subject characteristics.

|  | Non-BPD preterm group | BPD group | *p* value |
| --- | --- | --- | --- |
| NO. | 4 | 4 | / |
| Gender (Male/ Female) | 4/1 | 4/1 | >0.05 |
| CA(w) | 33.96±0.38 | 33.57±0.16 | >0.05 |
| BW(g) | 1.28±0.32 | 1.21±0.07 | >0.05 |
| Chorioamnionitis | 0/4 | 0/4 | >0.05 |
| Early-onset Sepsis | 0/4 | 1/4 | <0.05 |
| Use of Surfactant | 1/4 | 4/4 | <0.05 |
| Steroids use within 3 days prior to sampling | 0/4 | 0/4 | >0.05 |
| Antibiotic use within 3 days prior to sampling | 0/4 | 0/4 | >0.05 |
| Current Respiratory Support | 0/4 | 4/4 | <0.0001 |
| Severity | NA | Severe: 2  Moderate: 1  Mild: 1 |  |

Corrected gestational age, CA; Birth weight, BW;

## Supplementary Table 2 FCM subject characteristics.

|  | Non-BPD preterm group | BPD group | *p* value |
| --- | --- | --- | --- |
| NO. | 5 | 7 | / |
| Gender (Male/ Female) | 5/1 | 7/1 | >0.05 |
| CA(w) | 35.24±1.13 | 34.87±1.766 | >0.05 |
| BW(g) | 1.37±0.15 | 1.21±0.23 | >0.05 |
| Chorioamnionitis | 0/5 | 0/7 | >0.05 |
| Early-onset Sepsis | 0/5 | 3/7 | <0.05 |
| Use of Surfactant | 2/5 | 5/7 | <0.05 |
| Steroids use within 3 days prior to sampling | 0/5 | 0/7 | >0.05 |
| Antibiotic use within 3 days prior to sampling | 0/5 | 0/7 | >0.05 |
| Current Respiratory Support | 0/5 | 7/7 | <0.0001 |
| Severity | NA | Severe: 5  Moderate: 1  Mild: 1 |  |

Corrected gestational age, CA; Birth weight, BW;

## Supplementary Table 3 Human FCM antibody information.

| Specificity | Label | Clone | Manufacturer | Cat. No |
| --- | --- | --- | --- | --- |
| CD3 | Pe-cy7 | 0KT3 | TONBO biosciences | C0037021022603 |
| CD4 | BUV496 | SK3 | BD | 612936 |
| CD8 | Percp-cy5.5 | 0KT8 | TONBO biosciences | C0086073018653 |
| CD11b | BUV395 | M1/70 | BD | 563553 |
| CD11c | BV711 | 3.9 | BioLegend | 301630 |
| CD19 | PE-cy5 | HIB19 | TONBO biosciences | C0199052919533 |
| CD14 | APC | 61D3 | TONBO biosciences | C0149030422203 |
| CD15 | BV605 | W6D3 | BioLegend | 323032 |
| CD25 | FITC | BC96 | BioLegend | 302604 |
| CD127 | BV421 | A019D5 | BioLegend | 351310 |
| CD27 | BV650 | L128 | BD | 563228 |
| CD38 | PE | HB7 | TONBO biosciences | C0388061522503 |
| CD56 | PE-CF594 | 5.1H11 | BioLegend | 362544 |
| CD45RA | BV510 | HI100 | BD | 563031 |
| CCR7 | APC-R700 | 3D12 | BD | 565868 |
| HLA-DR | APC-cy7 | L243 | TONBO biosciences | C995207022253 |
| TNF-α | APC-cy7 | MAb11 | BioLegend | 502943 |
| IL-6 | PE | MQ2-13A5 | BioLegend | 501106 |
| IL-1β | FITC | H1b-98 | BioLegend | 511705 |

## Supplementary Table 4 Mouse FCM antibody information.

| Specificity | Label | Clone | Manufacturer | Cat. No |
| --- | --- | --- | --- | --- |
| CD3e | PE-cy5 | 145-2C11 | eBioscience | 15-0031-82 |
| CD4 | BV786 | RM4-5 | eBioscience | 417-0042-80 |
| CD8A | BV650 | 53-6.7 | eBioscience | 416-0081-82 |
| CD11b | BV605 | M1/70 | eBioscience | 406-0112-82 |
| CD11c | AF594 | N418 | Biolegend | 117346 |
| CD19 | AF700 | ID3 | eBioscience | 56-0193-82 |
| CD45 | BV510 | 30-F11 | Biolegend | 103138 |
| CD49b | Pe-cy7 | DX5 | eBioscience | 25-5971-82 |
| Gr-1 | APC | RB6-8C5 | Biolegend | 108470 |
| MHC-II | BV421 | M5/114.15.2 | eBioscience | 404-5321-82 |
| F4/80 | PE | BM8 | Biolegend | 111604 |
| Ly-6C | FITC | HK1.4 | Biolegend | 128006 |
| Ly-6G | AF780 | 1A8-Ly6g | eBioscience | 56-9668-82 |

## Supplementary Table 5 Primers for RT-PCR.

| Gene Name | Forward Primer | Reverse Primer |
| --- | --- | --- |
| β-actin | GGCTGTATTCCCCTCCATCG | CCAGTTGGTAACAATGCCATGT |
| Il6 | AGTTGCCTTCTTGGGACTGA | TCCACGATTTCCCAGAGAAC |
| Tnf-α | GGTGCCTATGTCTCAGCCTCTT | GCCATAGAACTGATGAGAGGGAG |
| Tnfrsf14 | CCAGGCTACTTCTGTGAGAACC | CAGTCAGCACATACAGTGTCCTG |
