# Supplementary material for: Single‐cell RNA sequencing of peripheral blood mononuclear cells from bronchopulmonary dysplasia
Source: Clin Transl Med. 2025 Mar 17;15(3):e70276. doi: 10.1002/ctm2.70276 (PMC11913593; doi:10.1002/ctm2.70276)
Supplement: Supplementary file 8 — Supporting Information [file CTM2-15-e70276-s004.pdf]

A

C

B

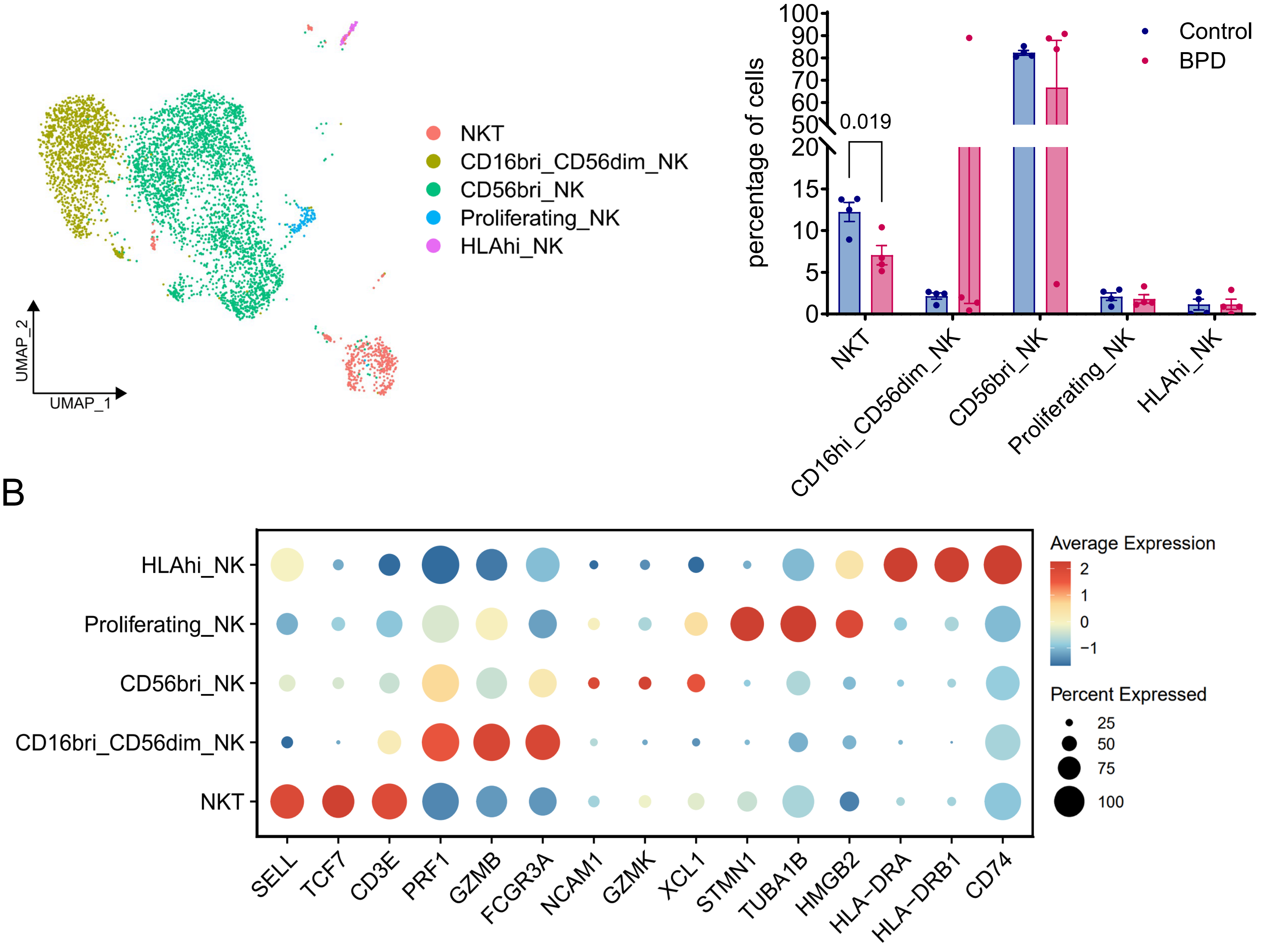

D

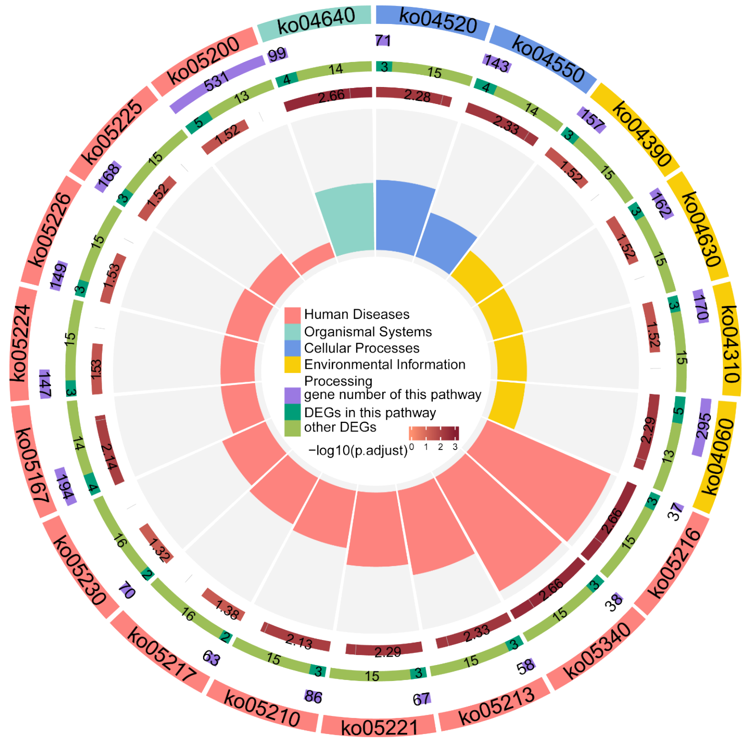

ID	Description
ko04640	Hematopoietic cell lineage
ko04520	Adherens junction
ko04550	Signaling pathways regulating pluripotency of stem cells
ko04390	Hippo signaling pathway
ko04630	JAK-STAT signaling pathway
ko04310	Wnt signaling pathway
ko04060	Cytokine-cytokine receptor interaction
ko05216	Thyroid cancer
Ko05340	Primary immunodeficiency
Ko05213	Endometrial cancer
Ko05221	Acute myeloid leukemia
ko05210	Colorectal cancer
ko05217	Basal cell carcinoma
ko05226	Gastric cancer
ko05225	Hepatocellular carcinoma
ko05200	Pathways in cancer
ko05230	Central carbon metabolism in cancer
ko05167	Kaposi sarcoma-associated herpesvirus infection
ko05224	Breast cancer
