# Supplementary material for: Single‐cell RNA sequencing of peripheral blood mononuclear cells from bronchopulmonary dysplasia
Source: Clin Transl Med. 2025 Mar 17;15(3):e70276. doi: 10.1002/ctm2.70276 (PMC11913593; doi:10.1002/ctm2.70276)
Supplement: Supplementary file 9 — Supporting Information [file CTM2-15-e70276-s009.pdf]

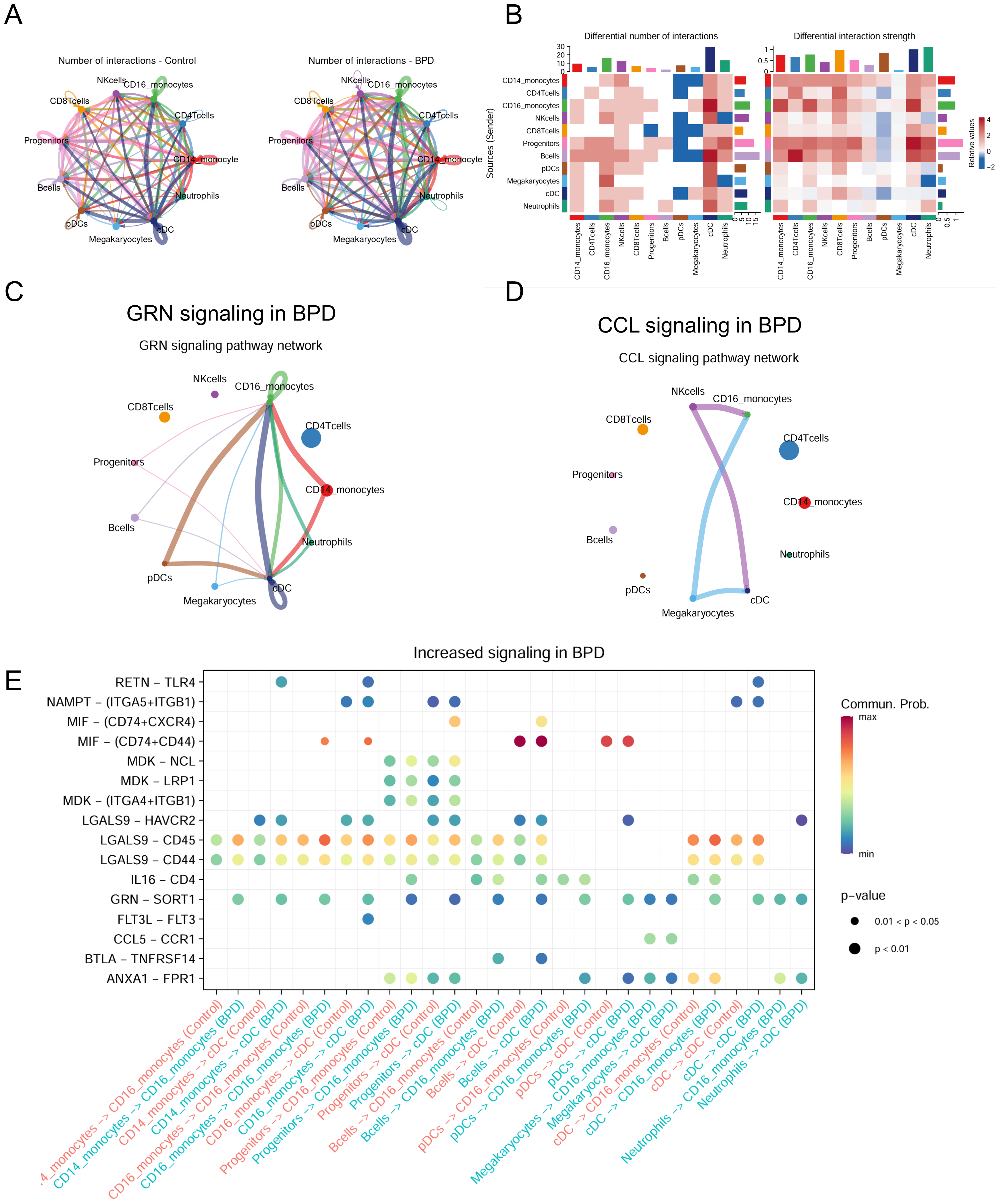
A
Number of interactions - Control
Number of interactions - BPD
B
Differential number of interactions
Differential interaction strength
Sources (Sender)
Relative values
CD14_monocytes
CD4Tcells
CD16_monocytes
NKcells
CD8Tcells
Progenitors
Bcells
pDCs
Megakaryocytes
cDC
Neutrophils
C
GRN signaling in BPD
GRN signaling pathway network
D
CCL signaling in BPD
CCL signaling pathway network
E
Increased signaling in BPD
RETN – TLR4
NAMPT – (ITGA5+ITGB1)
MIF – (CD74+CXCR4)
MIF – (CD74+CD44)
MDK – NCL
MDK – LRP1
MDK – (ITGA4+ITGB1)
LGALS9 – HAVCR2
LGALS9 – CD45
LGALS9 – CD44
IL16 – CD4
GRN – SORT1
FLT3L – FLT3
CCL5 – CCR1
BTLA – TNFRSF14
ANXA1 – FPR1
Commun. Prob.
max
min
p–value
0.01 < p < 0.05
p < 0.01
CD14_monocytes -> CD16_monocytes (BPD)
CD14_monocytes -> cDC (Control)
CD14_monocytes -> cDC (BPD)
CD16_monocytes -> CD16_monocytes (Control)
CD16_monocytes -> CD16_monocytes (BPD)
CD16_monocytes -> cDC (Control)
CD16_monocytes -> cDC (BPD)
Progenitors -> CD16_monocytes (Control)
Progenitors -> CD16_monocytes (BPD)
Progenitors -> cDC (Control)
Progenitors -> cDC (BPD)
Bcells -> CD16_monocytes (Control)
Bcells -> CD16_monocytes (BPD)
Bcells -> cDC (Control)
Bcells -> cDC (BPD)
pDCs -> CD16_monocytes (Control)
pDCs -> CD16_monocytes (BPD)
pDCs -> cDC (Control)
pDCs -> cDC (BPD)
Megakaryocytes -> CD16_monocytes (BPD)
Megakaryocytes -> cDC (BPD)
cDC -> CD16_monocytes (Control)
cDC -> CD16_monocytes (BPD)
cDC -> cDC (Control)
cDC -> cDC (BPD)
Neutrophils -> CD16_monocytes (BPD)
Neutrophils -> cDC (BPD)
